# MiRNA-15b and miRNA-125b are associated with regional Aβ-PET and FDG-PET uptake in cognitively normal individuals with subjective memory complaints

**DOI:** 10.1038/s41398-020-01184-8

**Published:** 2021-01-27

**Authors:** Andrea Vergallo, Simone Lista, Yuhai Zhao, Pablo Lemercier, Stefan J. Teipel, Marie-Claude Potier, Marie-Odile Habert, Bruno Dubois, Walter J. Lukiw, Harald Hampel, for the INSIGHT-preAD study group, Hovagim Bakardjian, Habib Benali, Hugo Bertin, Joel Bonheur, Laurie Boukadida, Nadia Boukerrou, Enrica Cavedo, Patrizia Chiesa, Olivier Colliot, Bruno Dubois, Marion Dubois, Stéphane Epelbaum, Geoffroy Gagliardi, Remy Genthon, Marie-Odile Habert, Harald Hampel, Marion Houot, Aurélie Kas, Foudil Lamari, Marcel Levy, Simone Lista, Christiane Metzinger, Fanny Mochel, Francis Nyasse, Catherine Poisson, Marie-Claude Potier, Marie Revillon, Antonio Santos, Katia Santos Andrade, Marine Sole, Mohmed Surtee, Michel Thiebaut de Schotten, Andrea Vergallo, Nadjia Younsi, Bruno Dubois, the Alzheimer Precision Medicine Initiative (APMI), Mohammad Afshar, Lisi Flores Aguilar, Leyla Akman-Anderson, Joaquín Arenas, Jesús Ávila, Claudio Babiloni, Filippo Baldacci, Richard Batrla, Norbert Benda, Keith L. Black, Arun L. W. Bokde, Ubaldo Bonuccelli, Karl Broich, Francesco Cacciola, Filippo Caraci, Giuseppe Caruso, Juan Castrillo, Enrica Cavedo, Roberto Ceravolo, Patrizia A. Chiesa, Massimo Corbo, Jean-Christophe Corvol, Augusto Claudio Cuello, Jeffrey L. Cummings, Herman Depypere, Andrea Duggento, Enzo Emanuele, Valentina Escott-Price, Howard Federoff, Maria Teresa Ferretti, Massimo Fiandaca, Richard A. Frank, Francesco Garaci, Hugo Geerts, Ezio Giacobini, Filippo S. Giorgi, Edward J. Goetzl, Manuela Graziani, Marion Haberkamp, Britta Hänisch, Karl Herholz, Felix Hernandez, Bruno P. Imbimbo, Dimitrios Kapogiannis, Eric Karran, Steven J. Kiddle, Seung H. Kim, Yosef Koronyo, Maya Koronyo-Hamaoui, Todd Langevin, Stéphane Lehéricy, Francisco Llavero, Jean Lorenceau, Alejandro Lucía, Dalila Mango, Mark Mapstone, Christian Neri, Robert Nisticò, Sid E. O’Bryant, Giovanni Palermo, George Perry, Craig Ritchie, Simone Rossi, Amira Saidi, Emiliano Santarnecchi, Lon S. Schneider, Olaf Sporns, Nicola Toschi, Pedro L. Valenzuela, Bruno Vellas, Steven R. Verdooner, Nicolas Villain, Kelly Virecoulon Giudici, Mark Watling, Lindsay A. Welikovitch, Janet Woodcock, Erfan Younesi, José L. Zugaza

**Affiliations:** 1 Sorbonne University, GRC n° 21, Alzheimer Precision Medicine (APM), AP-HP, Pitié-Salpêtrière Hospital, Boulevard de l’hôpital, F-75013 Paris, France; 2 Brain & Spine Institute (ICM), INSERM U 1127, CNRS UMR 7225, Boulevard de l’hôpital, F-75013 Paris, France; 3 Institute of Memory and Alzheimer’s Disease (IM2A), Department of Neurology, Pitié-Salpêtrière Hospital, AP-HP, Boulevard de l’hôpital, F-75013 Paris, France; 4 LSU Neuroscience Center, Louisiana State University Health Science Center, New Orleans, LA 70112 USA; 5 Department of Cell Biology and Anatomy, Louisiana State University Health Science Center, New Orleans, LA 70112 USA; 6 German Center for Neurodegenerative Diseases (DZNE), Rostock, Germany; 7 Department of Psychosomatic Medicine, University Medicine Rostock, Rostock, Germany; 8 ICM Institut du Cerveau et de la Moelle épinière, CNRS UMR7225, INSERM U1127, UPMC, Hôpital de la Pitié-Salpêtrière, 47 Bd de l’Hôpital, F-75013 Paris, France; 9 Sorbonne Université, CNRS, INSERM, Laboratoire d’Imagerie Biomédicale, F-75013 Paris, France; 10 Centre pour l’Acquisition et le Traitement des Images (www.cati-neuroimaging.com), Paris, France; 11 AP-HP, Hôpital Pitié-Salpêtrière, Département de Médecine Nucléaire, F-75013 Paris, France; 12 Alchem Biotech Research, Toronto, ON M5S 1A8 Canada; 13 Department of Ophthalmology, LSU Neuroscience Center, Louisiana State University Health Science Center, New Orleans, LA 70112 USA; 14 Department Neurology, LSU Neuroscience Center Louisiana State University Health Science Center, New Orleans, LA 70112 USA; 15 Ariana Pharmaceuticals, Paris, France; 16 Department of Anatomy and Cell Biology, McGill University, Montreal, QC Canada; 17 NeuroVision Imaging, Inc, Sacramento, CA USA; 18 Research Institute of the Hospital de Octubre (“imas”), Madrid, Spain; 19 Centro de Biología Molecular Severo Ochoa (CSIC-UAM), Madrid, Spain; 20 Department of Physiology and Pharmacology “Vittorio Erspamer”, Sapienza University of Rome, Rome, Italy; 21 Department of Clinical and Experimental Medicine, University of Pisa, Pisa, Italy; 22 Roche Diagnostics International, Rotkreuz, Switzerland; 23 Biostatistics and Special Pharmacokinetics Unit/Research Division, Federal Institute for Drugs and Medical Devices (BfArM), Bonn, Germany; 24 Department of Neurosurgery, Maxine Dunitz Neurosurgical Research Institute, Cedars-Sinai Medical Center, Los Angeles, CA USA; 25 Discipline of Psychiatry, School of Medicine and Trinity College Institute of Neuroscience (TCIN), Trinity College Dublin, Dublin, Ireland; 26 Federal Institute for Drugs and Medical Devices (BfArM), Bonn, Germany; 27 Unit of Neurosurgery, Azienda Ospedaliera Universitaria Senese, Siena, Italy; 28 Department of Drug Sciences, University of Catania, Catania, Italy; 29 Oasi Research Institute-IRCCS, Troina, Italy; 30 Genetadi Biotech S.L. Parque Tecnológico de Bizkaia, Derio, Bizkaia Spain; 31 Department of Neurorehabilitation Sciences, Casa Cura Policlinico, Milan, Italy; 32 CIC-1422, Centre National de Recherche Scientifique U 7225, Institut du Cerveau et de la Moelle Epinière, Assistance Publique Hôpitaux de Paris, Hôpital Pitié-Salpêtrière, Paris, France; 33 Department of Neurology and Neurosurgery, McGill University, H3G1Y6 Montreal, QC Canada; 34 Cleveland Clinic Lou Ruvo Center for Brain Health, Las Vegas, Nevada USA; 35 Department of Obstetrics and Gynaecology, Ghent University Hospital, Ghent, Belgium; 36 Department of Biomedicine and Prevention, University of Rome “Tor Vergata”, Rome, Italy; 37 2E Science, Robbio, Pavia Italy; 38 Medical Research Council Centre for Neuropsychiatric Genetics and Genomics, Cardiff University, Cardiff, UK; 39 Health Affairs CEO, UCI Health, University of California, Irvine, CA USA; 40 Institute for Regenerative Medicine, University of Zurich, Schlieren, Switzerland; 41 Department of Neurology, Translational Laboratory and Biorepository, University of California Irvine School of Medicine, Irvine, CA USA; 42 Siemens Healthineers North America, Siemens Medical Solutions USA, Inc, Malvern, PA USA; 43 In silico Biosciences, Computational Neuropharmacology, Berwyn, PA USA; 44 Department of Rehabilitation and Geriatrics, University Hospitals of Geneva, University of Geneva Medical School, Geneva, Switzerland; 45 Human Anatomy, Department of Translational Research and New Technologies in Medicine and Surgery, University of Pisa, Pisa, Italy; 46 Department of Medicine, University of California, San Francisco, CA United States; 47 Department of Physiology and Pharmacology “Vittorio Erspamer”, University of Rome “Sapienza”, Rome, Italy; 48 German Center for Neurodegenerative Diseases (DZNE), Bonn, Germany; 49 Division of Neuroscience and Experimental Psychology, University of Manchester, Manchester, UK; 50 Research & Development Department, Chiesi Farmaceutici, Parma, Italy; 51 Laboratory of Neurosciences, Intramural Research Program, National Institute on Aging, Baltimore, MD United States; 52 Foundational Neuroscience Center (FNC), AbbVie Neuroscience, Cambridge, MA USA; 53 MRC Biostatistics Unit, Cambridge Institute of Public Health, University of Cambridge, Cambridge, UK; 54 Department of Neurology, College of Medicine, Hanyang University, Seoul, Republic of Korea; 55 Department of Biomedical Sciences, Cedars-Sinai Medical Center, Los Angeles, CA USA; 56 Functional Neuromodulation, Ltd, Boston, MA USA; 57 Centre de NeuroImagerie de Recherche - CENIR, Institut du Cerveau et de la Moelle Épinière - ICM, Paris, France; 58 Achucarro Basque Center for Neuroscience, Science Park of the UPV/EHU, Leioa, Spain; 59 Institut de la Vision, INSERM, Sorbonne Universités, UPMC Univ Paris 06, UMR_S968, CNRS UMR7210, Paris, France; 60 Faculty of Sport Sciences, Universidad Europea de Madrid, Madrid, Spain; 61 Laboratory of Neuropharmacology, EBRI Rita Levi-Montalcini Foundation, Rome, Italy; 62 Department of Neurology, University of California Irvine School of Medicine, Irvine, CA USA; 63 Sorbonne University, CNRS UMR 8256, INSERM ERL U1164, Brain-C Lab, Paris, France; 64 University of North Texas Health Science Center, Fort Worth, TX USA; 65 College of Sciences, One UTSA Circle, The University of Texas at San Antonio, San Antonio, TX USA; 66 Centre for Clinical Brain Sciences, University of Edinburgh, Edinburgh, UK; 67 Department of Medicine, Surgery and Neurosciences, Unit of Neurology and Clinical Neurophysiology, Brain Investigation & Neuromodulation Lab. (Si-BIN Lab.), University of Siena, Siena, Italy; 68 Keck School of Medicine of the University of Southern California, Los Angeles, CA USA; 69 Department of Psychological and Brain Sciences, Indiana University, Bloomington, IN USA; 70 Systems Biology Department, University of Alcalá, Madrid, Spain; 71 Gérontopôle of Toulouse, Institute of Ageing, Toulouse University Hospital (CHU Toulouse), Toulouse, France; 72 TranScrip Partners, Reading, United Kingdom; 73 Department of Neurology and Neurosurgery, McGill University, Montreal, QC Canada; 74 Center for Drug Evaluation and Research, US Food and Drug Administration, Silver Spring, MD USA; 75 ITTM Solutions, Esch-sur-Alzette, Luxembourg

**Keywords:** Prognostic markers, Molecular neuroscience, Psychiatric disorders, Predictive markers

## Abstract

There is substantial experimental evidence for dysregulation of several microRNA (miRNA) expression levels in Alzheimer’s disease (AD). MiRNAs modulate critical brain intracellular signaling pathways and are associated with AD core pathophysiological mechanisms. First, we conducted a real-time quantitative PCR-based pilot study to identify a set of brain-enriched miRNAs in a monocentric cohort of cognitively normal individuals with subjective memory complaints, a condition associated with increased risk of AD. Second, we investigated the impact of age, sex, and the Apolipoprotein E *ε4* (*APOE ε4*) allele, on the identified miRNA plasma concentrations. In addition, we explored the cross-sectional and longitudinal association of the miRNAs plasma concentrations with regional brain metabolic uptake using amyloid-β (Aβ)-positron emission tomography (Aβ-PET) and ^18^F-fluorodeoxyglucose-PET (^18^F-FDG-PET). We identified a set of six brain-enriched miRNAs—miRNA-125b, miRNA-146a, miRNA-15b, miRNA-148a, miRNA-26b, and miRNA-100. Age, sex, and *APOE ε4* allele were not associated with individual miRNA abundance. MiRNA-15b concentrations were significantly lower in the Aβ-PET-positive compared to Aβ-PET-negative individuals. Furthermore, we found a positive effect of the miRNA-15b*time interaction on regional metabolic ^18^F-FDG-PET uptake in the left hippocampus. Plasma miRNA-125b concentrations, as well as the miRNA-125b*time interaction (over a 2-year follow-up), were negatively associated with regional Aβ-PET standard uptake value ratio in the right anterior cingulate cortex. At baseline, we found a significantly negative association between plasma miRNA-125b concentrations and ^18^F-FDG-PET uptake in specific brain regions. In an asymptomatic at-risk population for AD, we show significant associations between plasma concentrations of miRNA-125b and miRNA-15b with core neuroimaging biomarkers of AD pathophysiology. Our results, coupled with existing experimental evidence, suggest a potential protective anti-Aβ effect of miRNA-15b and a biological link between miRNA-125b and Aβ-independent neurotoxic pathways.

## Introduction

MicroRNAs (miRNAs)—a class of endogenous, single-stranded, 22-ribonucleotide-average-sized, non-coding RNAs—modulate post-transcriptional gene expression by either repressing translation or degrading their target messenger RNAs (mRNAs) after binding to the mRNAs 3′-untranslated region^1,2^. In the brain and central nervous system (CNS) miRNAs appear to act as single-discrete single-stranded RNA molecules or may be encapsulated into plasma membrane-derived exosomes (EXs) or extracellular microvesicles^3^.

In the brain—including the neocortex, hippocampus, and the limbic system—miRNAs regulate several intracellular signaling pathways involved in synaptic homeostasis, neuronal bioenergetics activity, and protein/lipid homeostasis^4,5^. In general, miRNA expression patterns are complex and dynamic; for instance, the natural miRNA abundance is subject to alterations during neural development and differentiation of the human brain and in the CNS during aging^6–8^.

Experimental models of aging and Alzheimer’s disease (AD), as well as in-human post-mortem and in vivo biomarker-based studies, indicate that the dysregulation of several miRNAs may influence AD pathophysiological mechanisms, including the amyloid-β (Aβ) pathway, tau pathology, brain immune, and inflammatory response, oxidative stress regulation, among others^7–16^.

Emerging data encourage further analysis and characterization of specific miRNAs that may be either downregulated or upregulated aberrantly in the prodromal and preclinical phases of AD^7,8,14–16^, where treatments with putative disease-modifying effect are more likely to be effective^17–19^.

Although abnormal miRNA patterns are exhaustively explored in AD cell culture models^20–25^ and patients’ tissue samples^26,27^, less information is available on extracellularly secreted miRNAs circulating in the peripheral blood, referred to as “circulating miRNAs”. They are either encapsulated by extracellular vesicles, such as EXs and microvesicles or bound to molecules such as the Argonaute protein or high-density lipoprotein cholesterol^28–32^.

In the present pilot study, we identified a set of six brain-enriched miRNAs (either brain-specific or highly expressed in the brain)—namely, miRNA-125b, miRNA-146a, miRNA-15b, miRNA-148a, miRNA-26b, and miRNA-100—in the INISGHT-preAD study cohort, a monocentric cohort of cognitively normal individuals with subjective memory complaint (SMC), a condition associated with increased risk of sporadic AD^33–35^.

We sought to examine the impact of primary biological factors–age, sex, and apolipoprotein E (*APOE*) *ε4* allele—on plasma concentrations of the selected miRNA set. Subsequently, we performed an exploratory study, both cross-sectional and longitudinal, to investigate whether baseline concentrations of these six miRNAs are associated with established neuroimaging biomarkers of AD pathophysiology, namely (I) Aβ-positron emission tomography (Aβ-PET), at both global and regional level, and (II) ^18^F-fluorodeoxyglucose-PET (^18^F-FDG-PET) to assess neuronal metabolism.

## Materials and methods

### Study participants and cognitive assessment

Sixty individuals with SMC were recruited from the standardized, large-scale, observational, monocentric, French academic university-based “INveStIGation of AlzHeimer’s PredicTors in Subjective Memory Complainers” (INSIGHT-preAD) study^35^, that is part of the Alzheimer Precision Medicine Initiative (APMI) and its established Cohort Program (APMI-CP)^36^. Participants were enrolled at the Institute of Memory and AD (*Institut de la Mémoire et de la Maladie d’Alzheimer*, *IM2A*) at the Pitié-Salpêtrière University Hospital in Paris, France.

The main objective of the INSIGHT-preAD study is to explore the earliest preclinical stages of AD and their progression to incipient objective cognitive impairment, using comprehensive clinical parameters and multi-modal biomarkers.

In brief, the INSIGHT-preAD study includes 318 cognitively and physically normal Caucasian individuals, recruited from the community in the wider Paris area, France, aged 70 to 85, with SMC. The status of SMC was confirmed as follows: (I) participants gave an affirmative answer (“YES”) to both questions: “Are you complaining about your memory?” and “Is it a regular complaint that has lasted for more than 6 months?”; (II) participants showed intact cognitive functions based on the Mini-Mental State Examination score (MMSE, ≥ 27), Clinical Dementia Rating scale (CDR = 0), and Free and Cued Selective Rating Test (FCSRT, total recall score ≥41). Aβ-PET imaging investigation was performed at the baseline visit, as a mandatory inclusion criterion. Thus, all individuals enrolled in the study have SMC and are stratified as either positive or negative for brain Aβ deposition. At baseline, demographic, clinical data, and *APOE* genotype (See Supplementary material) were collected in all participants. Exclusion criteria were a history of neurological or psychiatric diseases, including depressive disorders.

The study was conducted in accordance with the tenets of the Declaration of Helsinki of 1975 and approved by the local Institutional Review Board at the participating center (Ethical approval number: 2013-Fev-13150). All participants or their representatives gave written informed consent for the use of their clinical data for research purposes.

### Blood sampling and collections of tube storage

Ten (10) mL of venous blood were collected in one BD Vacutainer^®^ tube (lithium heparin), which was employed for subsequent analyses. Blood samples were taken in the morning, after a 12-hour fast, handled in a standardized way, and centrifuged for 15 minutes at 2000 G-force at 4 °C. Per sample, plasma fraction was collected, homogenized, aliquoted into multiple 0.5 mL cryovial-sterilized tubes, and finally stored at –80 °C within 2 hours from collection.

Data for plasma concentrations of the six miRNAs were collected at participants’ enrollment (“baseline visit” or “M0”).

### Total RNA isolation from human plasma

Plasma samples from age- and sex-matched SMC individuals (*N* = 60), dichotomized according to the Aβ-PET imaging status—either positive (*N* = 30) or negative (*N* = 30)—were selected for the analysis. Samples were thawed slowly on ice, and total RNA was isolated from 200 μL of plasma using a miRNeasy Serum/Plasma Advanced Kit (Qiagen, Germantown, MD) according to the manufacturer’s instructions for liquid samples. To allow for normalization of sample-to-sample variation in RNA isolation, 3.5 μL of 1.6 × 10^8^ copies/μL of synthetic *Caenorhabditis elegans* miRNA cel-miRNA-39 (Qiagen) was added to each sample right after the addition of lysis buffer (buffer RPL), during the first steps of the RNA isolation protocol. RNA was, then eluted with 20 μL of H_2_O according to the manufacturer’s instructions.

### Measurement of plasma miRNAs concentration

The samples were treated with heparinase during the reverse transcription (RT) step, as previously described elsewhere^37^, to overcome the inhibition from residual heparin to downstream real-time quantitative PCR (qRT-PCR) reactions. In brief, 0.25 μL of RNA was reverse-transcribed using the TaqMan High-Capacity cDNA Reverse Transcription Kit and miRNA-specific RT stem-loop primers (Thermo Fisher Scientific, Waltham, MA) in a 15-μL RT reaction containing 0.25 μL of the RNA extract, 0.15 μL of 100 mM dNTP, 1.0 μL of MultiScribe™ reverse transcriptase (50 U/μL), 1.5 μL of 10× RT buffer, 0.19 μL of RNase inhibitor (20 U/μL), 3.0 μL of 5× miRNA-specific stem-loop RT primer, 8.4 μL of RNase-free water and 0.5 μL of *Bacteroides* Heparinase I (12 U/μL) (New England Biolabs, Ipswich, MA). The reaction mixture was first incubated at 37 °C for 1 h for heparinase digestion, followed by RT reactions at 16 °C for 30 min, at 42 °C for 30 min, and at 85 °C for 5 min, and then maintained at 4 °C. The complementary DNA (cDNA) products were stored at −20 °C until analysis.

For qRT-PCR, the RT products were diluted 3× prior to qRT-PCR. In all, 2 μL of the diluted cDNA product was employed as a template in a 20-μL reaction containing 1.0 μL of TaqMan miRNA Assay (Thermo Fisher Scientific), 7.67 μL of RNase-free water, and 10 μL of TaqMan™ Universal Master Mix II, with UNG (Thermo Fisher Scientific). The qRT-PCR was performed with Bio-Rad CFX96 real-time PCR system (Bio-Rad) at 95 °C for 10 min, followed by 40 cycles of 95 °C for 15 s and 60 °C for 1 min (see Supplementary materials). Data were then analyzed with Bio-Rad CFX Manager (Bio-Rad) with the automatic Ct setting for assigning baseline and threshold for Ct determination. All quantities of the plasma miRNAs are expressed as relative quantities based on the 2-∆∆Ct method in which the relative expression level of each specific miRNA was calculated using the 2-∆∆Ct method after normalization to the spiked cel-miRNA-39.

In essence, all significantly changed plasma miRNA levels reported in this study (out of every human plasma miRNA (*N* = 2650)) were quantified using both RNA sequencing methods and qRT-PCR, adding to the analytical strength and robustness for this type of investigation.

### miRNA-based study design

A two-step pilot study was carried out on plasma samples from age- and sex-matched SMC individuals (*N* = 10) recruited from the INSIGHT-preAD study and dichotomized according to the Aβ-PET imaging status, either Aβ-PET positive (*N* = 5) or Aβ-PET negative (*N* = 5). Using RNA sequencing methods (LC Sciences, LLC, Houston, TX, US), significant intergroup differences were found in the plasma miRNA molecular signature [miRNA-125b, miRNA-146a, miRNA-15b, miRNA-148a, miRNA-26b, and miRNA-100]. Then, to explore this six-miRNA panel, the first step pilot study was expanded (*N* = 60) by recruiting further age- and sex-matched SMC individuals of the same cohort, dichotomized as Aβ-PET imaging positive (*N* = 30) or negative (*N* = 30). Outliers were removed before performing association and comparison analysis. The removal of the outliers was based on the 1.5 IQR (interquartile range = Q3–Q1) rule: data points outside of the Q1–1.5 IQR and Q3+1.5 IQR are considered as outliers. Data distribution among data groups may vary, including the number of outliers. However, in most cases, no more than two data points were removed in each group.

### PET data acquisition and processing

Aβ-PET investigation was performed at the baseline visit (“M0”)—as mandatory inclusion criterion—and at two-year follow-up (“M24”).

Brain amyloid PET scans were acquired 50 minutes after injection of 370 MBq (10 mCi) of ^18^F-Florbetapir. Brain ^18^F-FDG scans were obtained 30 minutes after injection of 2 MBq/kg of 2-deoxy-2-(^18^F)fluoro-d-glucose (^18^F-FDG). All acquisitions were performed in a single session on a Philips Gemini GXL scanner and consisted of 3 × 5 minutes frames with a voxel size of 2 × 2 × 2 mm^3^ ^35^.

Reconstructed PET images are analyzed with a pipeline developed by the CATI team, a French neuroimaging platform (http://cati-neuroimaging.com), according to a method previously described^35,38^. For longitudinal analysis, the mean activity in supratentorial white matter (eroded with a radius of three), the pons and whole-cerebellum regions were used as a reference for individual voxel normalization in the partial volume effect corrected images, as previously suggested^39^.

Standard uptake value ratios (SUVR) were calculated for each of 12 bilateral cortical regions of interest (anterior and posterior cingulate, superior frontal, inferior parietal, middle temporal cortices, and precuneus), as well as the global average SUVR.

A threshold of 0.79 obtained from a cross-sectional pipeline previously published^35^ was used in the present study to dichotomize individuals in Aβ-PET positive and Aβ-PET negative. Correlation analyses were performed through a longitudinal pipeline deployed through a stepwise process, previously published by our group^40^.

The same image-assessment pipeline was applied to measure brain glucose metabolism on ^18^F-FDG-PET scans, in a separate session. Cortical metabolic indices were calculated through Automated Anatomical Labeling atlas—120 ROIs, from which we selected the bilateral anterior cingulate cortex, posterior cingulate cortex, inferior parietal lobe, precuneus, middle temporal cortex, and hippocampus, with the pons used as the reference region.

### Statistical analysis

The analyses were conducted on a subset (*N* = 60) of the INSIGHT-preAD cohort with at least one plasma miRNA measure. First, we performed a comparison between Aβ-PET-positive and Aβ-PET-negative individuals seeking for the difference in plasma miRNA concentrations. Given the non-Gaussian distribution of miRNAs concentrations, we performed the Kruskal–Wallis one-way analysis of variance on ranks test.

Then, we investigated the effects of age, sex, and *APOE ε4* allele carrier status on each plasma miRNA concentration at baseline, using linear models (LM).

Then, we tested the association between plasma miRNAs concentration, at baseline, and brain Aβ accumulation, measured in terms of baseline and longitudinal SUVR, at both global and regional levels. Brain regions were a priori selected based on knowledge of the early stages of AD. To this aim, we used linear mixed models (LMM) with random intercept adjusted for age, sex, and *APOE ε4* carrier status. To investigate the longitudinal association between plasma miRNAs concentration and brain Aβ accumulation, we tested the interaction effect between baseline miRNAs concentration and time (miRNAs*time) on SUVR changes, over a 2-year follow-up (“M0” and “M24”).

Then, using a similar approach, we tested the association between plasma baseline miRNAs concentration and both baseline and longitudinal brain hypometabolism, assessed via ^18^F-FDG-PET, at the regional level.

Given the non-Gaussian distribution of miRNAs concentrations, we carried out permutation tests to investigate the statistical significance of the model coefficients. This method is more appropriate than parametric tests for small samples and when the sampling distribution is unknown^41,42^. Moreover, differently from rank-sum tests, permutation tests allow for interpretation of the results as the difference of median between groups^43^. Finally, permutation tests do not require estimation of statistic values or degrees of freedom. Thus, only *P* values were reported. In the present study, 5000 permuted samplings were run to estimate *P* values.

The full set of statistical analyses was conducted using the R software, version 3.6.0. Permutation tests for LM and LMM were computed using the “pgirmess” and “predictmeans” libraries, respectively, both available at http://cran.r-project.org/web/packages. *P* values < 0.05 were considered significant in all statistical elaborations. Given the explorative character of the present study, no correction of *P* values for multiple comparisons was applied.

## Results

### Participant characteristics, Aβ-PET group comparisons for miRNA concentrations, and association with key biological factors

Sociodemographic features, *APOE ε4* allele frequencies, Aβ-PET groups, and plasma baseline concentrations of the six miRNAs of the individuals are reported in Table 1.

**Table 1:** Sociodemographic features, *APOE ε4* allele frequencies, and plasma concentrations of the six identified brain-enriched miRNAs.

	*N*	All individuals	Sex		*APOE ε4* allele	
			Male	Female	Carriers	Non-carriers
Age, mean ± SD	60	74.23 ± 2.56	74.67 ± 2.90	73.94 ± 2.31	73.54 ± 2.22	74.43 ± 2.64
miRNA15b	57	0.69 (0.30–2.30)	0.59 (0.29–1.84)	0.73 (0.33–2.76)	0.49 (0.28–0.77)	0.78 (0.33–2.39)
miRNA26b	54	0.50 (0.36–1.89)	0.45 (0.37–1.30)	0.59 (0.35–2.31)	0.39 (0.34–0.77)	0.78 (0.40–1.98)
miRNA100	53	0.74 (0.49–1.46)	1.03 (0.49–1.46)	0.70 (0.51–1.48)	0.57 (0.39–1.29)	0.82 (0.51–1.48)
miRNA125b	53	0.78 (0.58–1.11)	0.77 (0.61–1.04)	0.78 (0.54–1.12)	0.65 (0.47–0.79)	0.85 (0.59–1.12)
miRNA146a	53	0.49 (0.32–1.43)	0.47 (0.30–1.19)	0.70 (0.33–1.64)	0.40 (0.28–0.61)	0.79 (0.34–1.64)
miRNA148a	53	0.62 (0.34–1.35)	0.63 (0.36–1.37)	0.51 (0.34–1.33)	0.32 (0.27–0.70)	0.69 (0.36–1.41)

Plasma miRNAs are reported in terms of median (IQ) of concentration (relative quantity).

*APOE ε4* Apolipoprotein E *ε4* allele, *IQ* interquartile, *miRNA* microRNA, *N* complete observation, *SD* standard deviation.

**Fig. 1:**
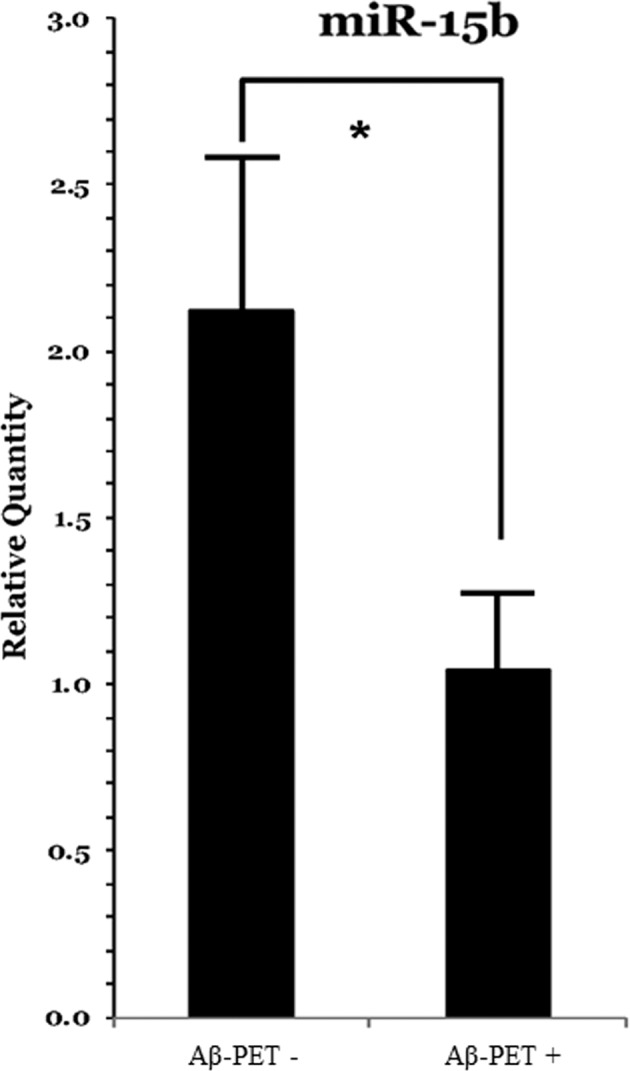
miRNA15b is significantly lower in the Aβ-PET negative subgroup compared with the Aβ-PET positive. *Kruskal–Wallis one-way ANOVA on ranks test; *p* value: 0.045 (see Table 1 for descriptive data). Plasma miR-15b concentration is reported in terms of relative quantity. *PET* positron emission tomography, *Aβ* amyloid beta, *n.s.* not significant, *miRNA* microRNA.

Among the miRNAs identified in the total sample of 60 individuals, only miRNA-15b was different between the two Aβ-PET subgroups, being significantly lower in the Aβ-PET-positive individuals than Aβ-PET-negative ones (see Table 2 and Fig. 1).

**Table 2:** Comparison of Aβ-PET subgroups for plasma concentrations of the six identified brain-enriched miRNAs.

	Aβ-	Aβ+	*p* value
miRNA15b	0.83 (0.42–3.14)	0.49 (0.22–1.54)	0.045*
miRNA26b	0.66 (0.41–1.87)	0.46 (0.32–1.88)	n.s.
miRNA100	1.03 (0.62–1.48)	0.59 (0.41–1.43)	n.s.
miRNA125b	0.76 (0.55–1.09)	0.83 (0.58–1.07)	n.s.
miRNA146a	0.75 (0.42–1.62)	0.35 (0.29–1.33)	n.s.
miRNA148a	0.62 (0.43–1.34)	0.51 (0.30–1.27)	n.s.

*Kruskal–Wallis one-way ANOVA on ranks test. Plasma miRNAs are reported in terms of median (IQ) of concentration (relative quantity).

*PET* positron emission tomography, *Aβ* amyloid beta, *n.s.* not significant, *miRNA* microRNA.

No association between plasma concentrations of the six miRNAs and age was found (*P* > 0.145). Plasma concentrations of all miRNAs did not significantly differ between males and females (*P* > 0.214).

Likewise, we did not observe a significant *APOE ε4* allele effect on plasma baseline miRNAs concentrations (*P* > 0.083).

### Effect of plasma baseline miRNAs concentrations on the rate of brain Aβ deposition

We investigated whether plasma baseline concentrations of each miRNA are associated with baseline (M0) and follow-up (M24) brain Aβ accumulation, at both the global and regional level.

At M0, we found a negative association between plasma miRNA-100 concentrations and Aβ-PET SUVR in the posterior cingulate cortex (left *P* = 0.046; right *P* = 0.049). A negative association was also observed between plasma miRNA-125b concentrations and Aβ-PET SUVR in the right anterior cingulate cortex (*P* = 0.047).

LMM also showed a significantly negative association of the miRNA-125b*time and miRNA-148a*time interactions with Aβ-PET SUVR in the right anterior cingulate cortex (*P* = 0.030 and *P* = 0.045, respectively), over a two-year follow-up (M0 and M24).

No plasma miRNA was associated with global Aβ-PET SUVR. We did not find any significant associations between regional Aβ-PET SUVR and plasma miRNA-15b, miRNA-26b, and miRNA-146a.

### Effect of plasma baseline miRNAs concentrations on brain metabolism

We investigated whether plasma baseline concentrations of each miRNA were associated with baseline (M0) and follow-up (M24) of the regional ^18^F-FDG-PET signal.

We found a significantly negative association between plasma miRNA-125b concentrations and ^18^F-FDG-PET signal in the following regions: posterior cingulate cortex (left *P* = 0.011; right *P* = 0.002), anterior cingulate cortex (left *P* = 0.006; right *P* = 0.014), left inferior parietal cortex (*P* = 0.048), middle temporal cortex (left *P* = 0.027; right *P* = 0.031), and left hippocampus (*P* = 0.019), at M0.

Plasma miRNA-148a concentrations negatively correlated with ^18^F-FDG-PET signal in the right inferior parietal cortex (*P* = 0.023) and precuneus (left *P* = 0.030; right *P* = 0.049), at M0.

LMM showed a positive miRNA-125b*time interaction in the right posterior cingulate cortex (*P* = 0.035), over a 2-year follow-up (M0 and M24).

A significantly negative miRNA-148a*time interaction was found in the following regions: right anterior cingulate cortex (*P* = 0.045), right inferior parietal cortex (*P* = 0.020), precuneus (left *P* = 0.036; right *P* = 0.037), and right middle temporal cortex (*P* = 0.041).

We found a positive effect of the miRNA-15b*time interaction in the left hippocampus (*P* = 0.021) and a negative miRNA-26b*time interaction in the left precuneus (*P* = 0.048).

No significant associations with ^18^F-FDG-PET signal were found for plasma miRNA-100 and miRNA-146a.

## Discussion

To our knowledge, this is the first study investigating the association of miRNA-based signatures in plasma with AD core neuroimaging biomarkers of the Aβ pathway and neuronal metabolism, in a cohort of cognitively normal individuals with SMC, a condition at increased risk for AD.

We observed no significant association between age, sex, *APOE ε4* status, and individual miRNA abundance or speciation, suggesting that miRNAs may represent an independent variable in specific neurological disease states. In similar studies analyzing the influence of the confounding factors age and sex on miRNA profiles from peripheral blood, it was found that very few single miRNAs remained significantly associated with age and sex after adjustment using multiple testing parameters^44^. This lack of significant association may also be a reflection of (I) the observation that many miRNA biomarkers show discrepant results in independent investigations of the same disease, and (II) the remarkable heterogeneity of miRNA complexity and expression patterns in neurological disorders, such as those observed across the entire AD continuum^45^.

We report significantly lower levels of miRNA-15b in Aβ-positive individuals than -negative ones.

Interestingly, plasma miRNA-15b, integrated into a panel of other miRNAs, was found specifically downregulated in AD patients compared with both normal controls^46,47^ and Parkinson’s disease patients^47^; it also positively correlated with cerebrospinal fluid (CSF) Aβ_1-42_ concentrations^47^. In another study, the profiling of exosomal miRNAs extracted from serum samples disclosed miRNA-15b downregulated expression in AD patients compared to healthy control individuals^48^.

Such evidence led to infer a potential link between miRNA-15b and protective dynamics counteracting the dysregulation of the Aβ pathway. This hypothesis is supported by studies conducted in SH-SY5Y/APPswe neuroblastoma cell lines that reported an association of miRNA-15b with: (I) inhibition of brain Aβ accumulation, by directly downregulating β-site amyloid precursor protein cleaving enzyme 1 (BACE1) protein expression or (I) attenuation of Aβ-induced secretion/expression of pro-inflammatory cytokines, by suppressing the pro-inflammatory nuclear factor kappa-light-chain-enhancer of activated B cells signaling pathway activation^49^. This experimentally inferred anti-Aβ effect alongside the statistical difference between our two Aβ-PET subgroups of individuals, suggested an association between miRNA-15b and regional Aβ-PET SUVRs that we did not find. Although there may be a neurobiological explanation, we cannot rule out a statistical Type II error related to the small sample size (see also below).

We also found a positive association between miRNA-15b concentrations and levels of neuronal metabolism (^18^F-FDG-PET SUVR) in the hippocampus. Although an interpretation of this finding is rather challenging, it is worth noting that miRNA-15b is known for its regulatory activity of cortical development and its involvement in the expansion and differentiation of cortical neural progenitor cells^50^.

We showed associations of miRNA-125b and miRNA-148a concentrations with Aβ-PET SUVR and miRNA-15b with ^18^F-FDG-PET signal in some early AD-related brain regions and the hippocampus.

In the present study, the negative baseline association between plasma miRNA-125b and miRNA-100 concentrations and Aβ-PET SUVR in the right anterior cingulate cortex and the bilateral posterior cingulate cortex indicate a potential modulatory role of these miRNAs on the Aβ pathway, tracked at the fibrils and plaques phase when investigated with the current Aβ-PET radiotracers. The negative association of miRNA-125b and miRNA-148a concentrations with baseline and follow-up Aβ-PET SUVR in the right anterior cingulate cortex strengthens such a hypothesis.

The negative associations between these three miRNAs (miRNA-125b, miRNA-100, and miRNA148a) and baseline and follow-up levels of Aβ-PET radiotracer uptake support a knowledge-based, albeit speculative, hypothesis that the miRNAs in question are linked to molecular mechanisms that inhibit Aβ deposition.

In an attempt to address our results from an overarching neurobiological standpoint, we outline that miRNA-125b is the miRNA more extensively investigated in the AD human brain and biofluids and experimental models of the disease as well. Studies conducted in primary mouse cortical neurons (MCN) and neuroblastoma Neuro2a (N2a) cells show that miRNA-125b binds to the BACE1 protein with an inhibitory effect on its expression levels and mitigates Aβ-induced neurotoxicity^51^. In particular, BACE1 protein acted as a target of miRNA-125b and was negatively modulated by this miRNA in both MCN and N2a cells. BACE1 restoration inhibited the effect of miRNA-125b on Aβ-induced neurotoxicity, thus indicating that miRNA-125b might repress AD development by reducing BACE1 expression. Moreover, the authors showed that toxic downstream effects of Aβ species, such as apoptotic rate, promotion of caspase-3 activity (crucial for neuronal apoptosis), and oxidative stress—were all decreased. The same study also reported that miRNA-125b and BACE1 mRNA serum expression levels were respectively reduced and increased in patients with sporadic AD compared to age-matched healthy individuals. A negative correlation between miRNA-125b and BACE1 mRNA serum expression levels was found in AD patients^51^.

CSF and blood (serum)-based studies reported down-regulation of miRNA-125b in AD patients compared with a cohort of non-inflammatory and inflammatory neurological disease controls^52^ and to healthy controls^53^. The same result was reported in two independent studies conducted in either CSF or plasma^54^.

By contrast, our miRNA-125b-related result seems to counteract a previous post-mortem study reporting that miRNA-125b was significantly and distinctively upregulated in AD temporal lobe neocortex, potentially contributing to the neurodegenerative process of the disease^55^. Another study reported that miRNA-125b is also overexpressed in the hippocampus in AD late stages (Braak stages V, VI)^56^.

The studies mentioned above support the negative (five out of eight regions, with only one region proving positively associated and counteracting this trend of associations) association we found between miRNA-125b and ^18^F-FDG-PET indexes in multiple brain regions involved in early AD. This finding is in line with experimental studies that showed an association between miRNA-125b and synaptic toxicity in cultured hippocampal neurons^57^ as well as between miRNA-125b overexpression and impairment of synaptic remodeling and transmission coupled with inhibition of the long-term potentiation (LTP) activation^58^.

Moreover, miRNA-125b, one of the most highly expressed microRNAs in the human brain and retina, was associated with kinase/phosphatase activity inbalance, increased tau phosphorylation and its downstream perturbation of the synaptic activity^59^.

In the present study, the absence of converters from SMC to mild cognitive impairment (MCI) (or even overt dementia) does not allow drawing any meaningful biological or clinical conclusion on the role of miRNA-125b in AD; however, our PET-based results coupled with the existing literature suggest that miRNA125b may be linked to Aβ-independent neurotoxic pathways. The limitations of the present exploratory study will need to be overcome in future investigations with longer follow-up and higher rates of conversion to MCI and AD dementia to untangle the role, either mono-dimensional or dualistic, of miRNA-125b.

There is no extensive experimental evidence to explain the unprecedented association we here report for miRNA-148a and miRNA-100 with biomarkers charting the Aβ pathway and neuronal metabolism. However, these miRNAs have been previously investigated in AD, with the former upregulated in AD patients^60^, and miRNA-100 found lower in AD versus non-demented controls^61^.

Neurobiological studies pointed out miRNA-148a as upregulated in the hippocampus of 3xTgAD mice versus their age-matched controls^62^, and it was highly concentrated in synapses^63^ and downregulated the expression of Ca^2+^/calmodulin-dependent protein kinase IIα^64^, a key protein for Calcium homeostasis. Calcium dysregulation has been demonstrated to potentially occur upstream to brain proteinopathies with Aβ monomers production and tau hyperphosphorylation that can be triggered by a sustained increase of cytosolic Calcium concentrations over basal levels^65^. From a functional standpoint, miRNA-148a upregulation was associated with impairment of LTP that underlies memory dysfunction in AD^64^.

Regarding the positive association found between miRNA-148a and ^18^F-FDG-PET SUVR, at both baseline and follow-up, the interpretation of this data is rather challenging given the lack of experimental evidence about this miRNA and neuronal functions.

### Limitations

The study presents some caveats that need to be addressed. First, the restricted sample size of the first step of the pilot study (*N* = 10) potentially limited the number of miRNAs dysregulated that were detected between groups. Moreover, the relatively small sample size of the second step pilot study (*N* = 60) limited the generalizability of our conclusions. The absence of converters to MCI or even dementia stage constrains the clinical conclusions that can be drawn. Future studies in larger cohorts of SMC individuals, with extensive follow-up, are needed to corroborate or disconfirm the findings of the present pilot data.

We performed a priori investigation on selected brain regions rather than executing a whole-brain exploratory strategy. Although the latter approach might have contributed to untangle patterns of brain Aβ accumulation and synaptic activity, we could not run it owing to statistical reasons (i.e., statistical power).

Finally, our investigation was exclusively performed on a sample of Caucasian individuals; hence, replicating our findings in different ethnic populations would be remarkable.

To increase the clinical meaningfulness of the present exploratory study results, we intend to carry out a subsequent study in a larger population of INSIGHT-preAD cognitively healthy individuals, possibly employing an independent validation cohort, and including (i) blood-based biomarkers charting AD pathophysiological alterations and (ii) longer follow-up of neuroimaging and cognitive measures. We set out to explore whether plasma miRNA can predict AD clinical-biological trajectories; thus, representing a candidate non-invasive and accessible screening and prognostic tool. If our results were corroborated, plasma miRNA could be integrated into a blood-based biomarker matrix for precision medicine-oriented large-scale investigation of cognitively healthy individuals at increased risk of developing rapid AD pathophysiological and cognitive impairment, a highly suitable population for disease-modifying therapeutic approaches when these will be available.

## Conclusions

This is the first exploratory study reporting significant associations between plasma concentrations of a signature of miRNAs we identified and AD core neuroimaging biomarkers of the Aβ pathway and neuronal metabolism in a cohort of SMC individuals, a condition associated with increased risk of AD. In particular, our study provides preliminary evidence in vivo about the potential role of miRNAs -125b and -15b, as candidate miRNA biomarker of AD pathophysiology.

In summary, our findings are consistent with the endevour of generating insights about the roles that circulating miRNAs may play for AD pathophysiology. Results turning out of this research line may inform precision-medicine oriented pharmacological trials that take into account the entire biological profile of the single patient. Such an approach could be then generalized to the broad spectrum of neurodegenerative diseases by developing pathway-based therapies in biologically ideal individuals, in line with the precision medicine paradigm shift.

## Supplementary information

Supplemental Material
