# Supplementary material for: MiRNA-15b and miRNA-125b are associated with regional Aβ-PET and FDG-PET uptake in cognitively normal individuals with subjective memory complaints
Source: Transl Psychiatry. 2021 Jan 27;11:78. doi: 10.1038/s41398-020-01184-8 (PMC7840941; doi:10.1038/s41398-020-01184-8)
Supplement: Supplementary file 1 — Supplemental Material [file 41398_2020_1184_MOESM1_ESM.docx]

**SUPPLEMENTARY MATERIAL**

**METHODS**

**Absolute quantification of plasma miRNAs using the standard curve method**

**miRNA-15b**


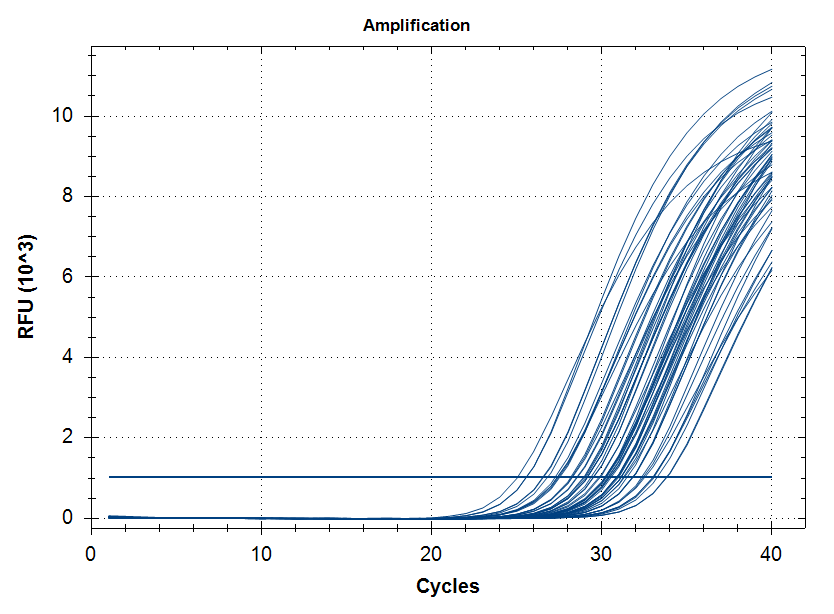


**miRNA-125b**


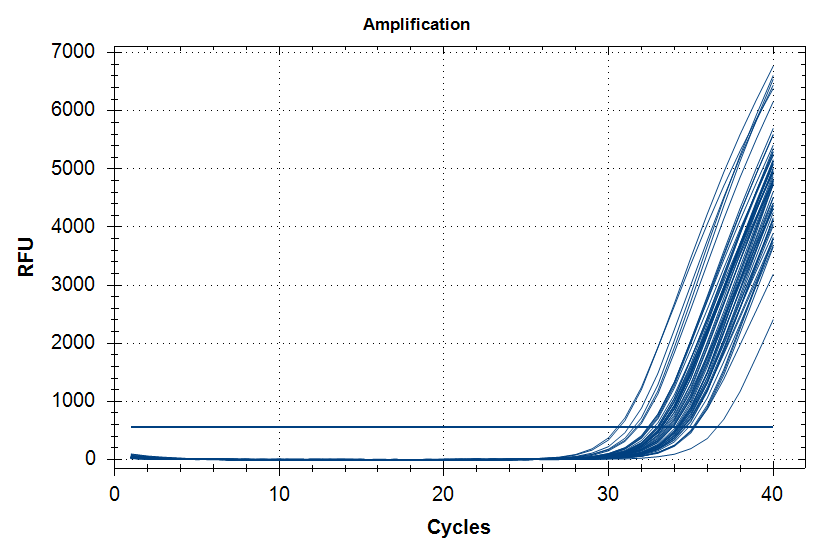


**miRNA-26b**


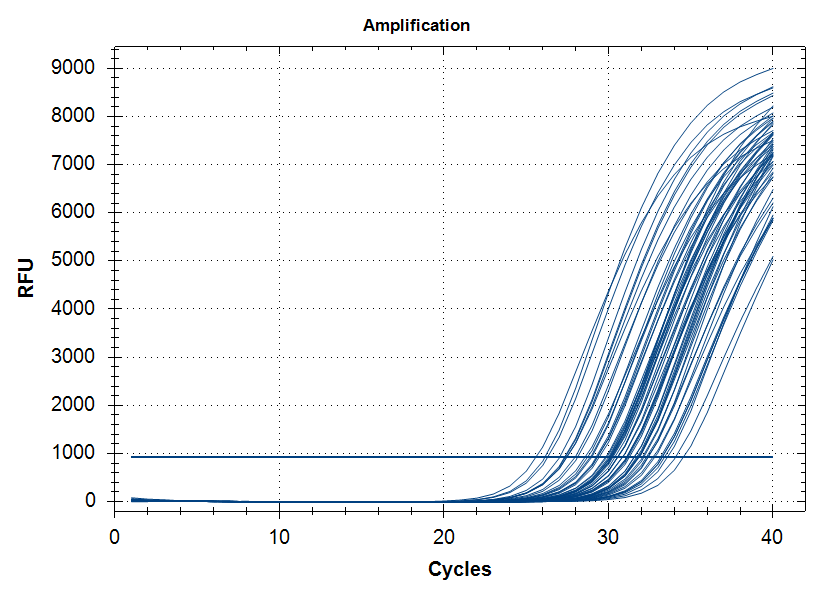


**miRNA-100**


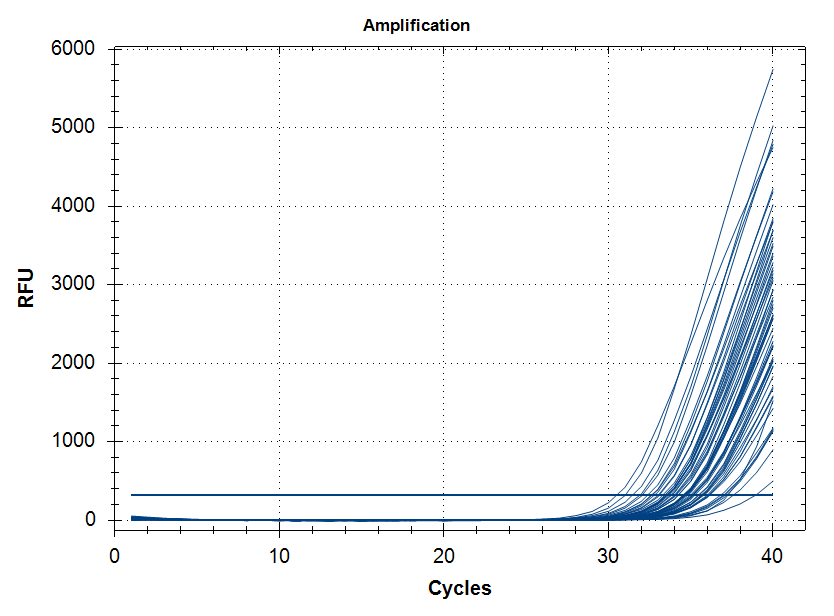


**miRNA-146a**


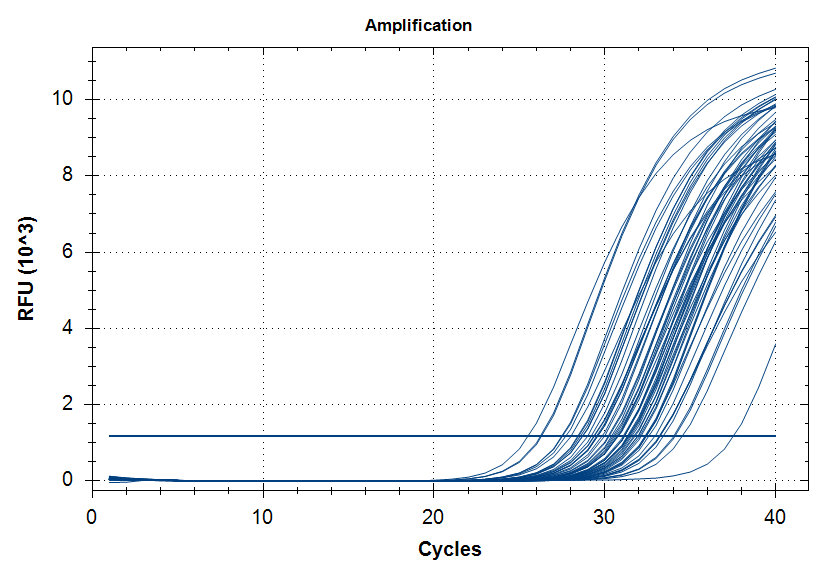


**miRNA-148a**


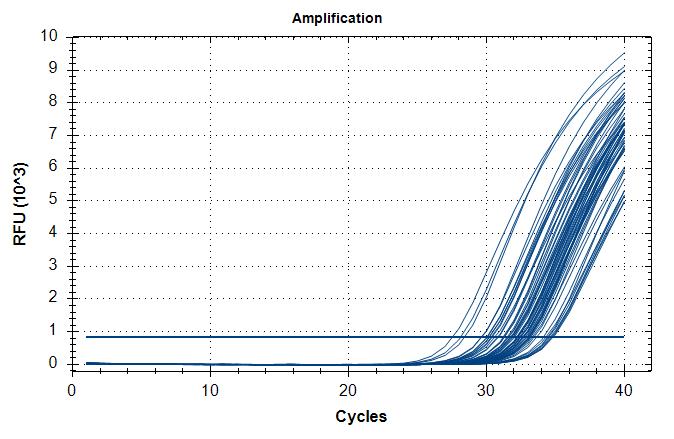


*Abbreviations:* RFU: relative fluorescence units; miRNA: microRNA

*Note:* The relative expression level (relative quantity, RQ) of each specific miRNA was assessed through comparative CT method that compares the Ct value of one target gene to another, i.e., an internal control or reference gene (e.g., housekeeping gene)—in a single sample. In the repsent study, All quantities of the plasma miRNAs are expressed as relative quantities based on the 2-∆∆Ct method in which the relative expression level of each specific miRNA was calculated using the 2-∆∆Ct method after normalization to the spiked cel-miRNA-39.

***APOE* genotype**

DNA was extracted from frozen blood samples of participants applying the 5Prime ArchivePure DNA purification system. The *APOE* genotypes were determined using Sanger method. Exon 4 from *APOE* gene holding the SNP related to the *ε3/ε4* alleles was amplified using PCR with the following primers: APOE sense, 5’-TAAGCTTGGCACGGCTGTCCAAGGA-3’; APOE antisense, 5’-ACAGAATTCGCCCCGGCCTGGTACAC-3’. For each sample, the reaction mixture (50µl) contained 200ng of genomic DNA, 10µl PCR Flexi buffer (5x), 3µl MgCl2 (25mM), 1µl dNTPs (10mM), 1µl of each forward and reverse primers (10µM), and 0.25µl GO Taq DNA polymerase (Promega). The cycling program was carried out after a preheating step at 95°C for 2 minutes and 35 cycles of denaturation at 95°C for 1 minute, annealing at 68°C for 1 minute and extension at 72°C for 1 minute. The amplified fragments were then purified and sequenced using the same primers.
